# Audiological Outcome of Tympanoplasty With Mastoidectomy in Middle-Aged and Younger Patients With Chronic Otitis Media

**DOI:** 10.7759/cureus.75121

**Published:** 2024-12-04

**Authors:** Saurabh Saini, Saquib Reyaz Khan, Sabeeh Beig, Vanshika Singhal

**Affiliations:** 1 Otolaryngology - Head and Neck Surgery, Varun Arjun Medical College & Rohilkhand Hospital, Shahjahanpur, IND; 2 Otolaryngology - Head and Neck Surgery, Jawaharlal Nehru Medical College and Hospital, Aligarh, IND

**Keywords:** audiological gain, chronic otitis media (com), middle-aged patients, otorhinolaryngology, pure tone audiometry, younger patients

## Abstract

Objective: Chronic otitis media (COM) is characterized by chronic, intermittent, or persistent discharge through a perforated tympanic membrane. In this study, we aimed to evaluate the postoperative audiological outcomes in middle-aged patients compared to younger patients who underwent tympanoplasty with mastoidectomy via post-auricular approach for the treatment of COM.

Methods: This prospective interventional study included patients admitted in wards from August 2017 to January 2019 at the Department of Otorhinolaryngology, Jawaharlal Nehru Medical College and Hospital, Aligarh, India. A total of 70 patients diagnosed with COM were enrolled and divided into two groups. Group A (middle-aged COM group) included patients 41 to 60 years of age and Group B (younger COM group) consisted of patients 21 to 40 years of age. Patients were followed up for a period of approximately six to nine months to assess the audiological status evaluated at three, six, and nine months after surgery.

Results: It was found that after three, six, and nine months post-surgery, the difference in the mean audiological gain was not statistically significant, with p-values at 0.3034, 0.3271, and 0.2923, respectively. The audiological gain demonstrated a slight decrease over time in both groups.

Conclusion: Audiological improvement, measured as the mean audiological gain, demonstrated a slight decrease over time in both groups. However, there was no significant difference in the improvement of the air-bone gap between middle-aged and younger patients.

## Introduction

Chronic otitis media (COM), a common condition in otorhinolaryngology, is characterized by chronic, intermittent, or persistent discharge through a perforated tympanic membrane (TM) [1]. COM is clinically characterized as an inflammatory condition associated with otorrhoea and tympanic membrane perforation in some cases [2]. In COM, tympanic membrane perforation and ossicular chain erosion cause defective middle ear function leading to hearing loss. The proposed mechanism for erosion is chronic middle ear inflammation resulting from the overproduction of cytokines TNF alpha, interleukin-2, fibroblast growth factor, and platelet-derived growth factor, which promote hypervascularization, osteoclast activation, and bone resorption causing ossicular damage [1].

The human ear can recognize sound waves in a frequency range of 20-20,000 Hz. Human speech frequencies are in the range of 500-4000 Hz, and the decibel level for a normal conversation is between 45 and 60 dB. The degree of hearing loss depends on the size of perforation in the tympanic membrane, ossicular chain, and inner ear. Pure tone audiometry helps to identify hearing thresholds of an individual based on the intensity and pitch of the sound waves. Since hearing loss is the most common complication associated with COM, which affects day-to-day activity and the quality of life of an individual, an attempt is made to analyze the hearing loss by audiometry in patients with COM [3,4]. The goals of any surgery for COM include the creation of a dry, safe ear and the maximal preservation or restoration of hearing [5]. The management of chronic otitis media, with or without cholesteatoma, is cortical mastoidectomy with TM perforation repair and/or ossicular chain repair, which is considered the treatment of choice for active chronic otitis media [6].

The aim of this study was to evaluate the postoperative audiological outcomes in middle-aged patients compared to younger patients who underwent tympanoplasty with mastoidectomy via post-auricular approach for the treatment of COM. Additionally, we compared these postoperative results with those of younger patients, providing insights into the effectiveness and safety of the procedure across different age groups.

## Materials and methods

This study was conducted at the Department of Otorhinolaryngology, Jawaharlal Nehru Medical College and Hospital, Aligarh Muslim University, Aligarh, after obtaining ethical clearance from the Institutional Ethics Committee (approval no. 652/FM, dated July 17, 2017). A total of 70 patients, admitted in wards from August 2017 to January 2019, suffering from chronic otitis media with or without cholesteatoma who underwent tympanoplasty with mastoidectomy were included. The patients were divided into two groups: Group A (middle-aged COM group) that included patients 41 to 60 years of age and Group B (younger COM group) with patients 21 to 40 years of age. The sample size was calculated using the formula, n = 2(SD)^2^ (Zα+ Zβ)^2^/d^2^, for comparison of means (audiological gain) between two groups (middle-aged COM group and younger COM group). Based on this calculation, the sample size was determined to be 35 in each group. Here, the pooled standard deviation (SD) was taken as 3.10 from a previous study, Zα was 1.96 (corresponding to a 95% confidence interval), Zβ was 0.84 (indicating 80% power), and the minimum detectable difference (d) was 2 dB based on previous research findings [7]. The study aimed to compare the audiological gain between the two groups, i.e., the middle-aged COM group and the younger COM group. All the patients were subjected to detailed history taking, a general as well as systemic examination, which included clinical examination of the ear, nose, and throat, and a complete otological evaluation done to assess the exact nature and extent of the disease.

Inclusion and exclusion criteria

Patients with chronic otitis media with isolated conductive hearing loss undergoing tympanoplasty with mastoidectomy, patients between 21 to 60 years of age, and patients with appropriate cochlear reserve were included in the study. Patients with medical contraindications to surgery, COM with intracranial complications, and cases requiring revision surgery for residual disease were excluded.

Follow-up

Patients were followed up for a period of approximately six to nine months to assess postoperative complications and disease recurrence. Audiological status was evaluated at three, six, and nine months after surgery.

Comparisons between middle-aged and younger COM groups

To compare the postoperative results of tympanoplasty with mastoidectomy between middle-aged and younger-aged COM groups, we compared the hearing improvements in the two COM groups. We calculated mean hearing levels by averaging the hearing thresholds at 500, 1000, 2000 and 4000 Hz (to yield the four-tone pure tone average) after three, six, and nine months of surgery. Then we compared the preoperative and postoperative air-bone (AB) gaps between the middle-aged and younger COM groups. Additionally, we compared postoperative complications between the two groups. Postoperative results of tympanoplasty with mastoidectomy were evaluated in terms of average hearing gain.

Statistical analysis

Data were analyzed using IBM SPSS Statistics, version 20 (IBM Corp., Armonk, NY). Continuous variables were expressed as means ± standard deviations, and categorical variables were expressed as frequencies and percentages. The unpaired t-test and chi-square test were used to compare the outcomes between the two groups. A p-value of <0.05 was considered statistically significant [8].

## Results

The demographic profile of the participants is summarized in Table 1. The mean age was 48.23 years (SD = 5) in the middle-aged group and 29.66 years (SD = 5) in the younger group. The groups were comparable in terms of gender distribution, with a slight predominance of males in both groups. Rural participants were more prevalent in both groups. In the middle-aged group, there were 19 male and 16 female patients, with a male-female ratio of 1.19:1. In the younger age group, there were 21 male and 14 female patients, with a male-to-female ratio of 1.5:1 (Table 1).

**Table 1:** Demographic profile of participants

Demographic	Middle-aged group	Younger group	p-value
Mean age (years)	48.23	29.66	0.00001
Age range (years)	41-60	21-40	-
Male	19	21	0.0432
Female	16	14	0.7549
Rural (%)	57	66	0.8538
Urban (%)	43	34	0.5687

Ear discharge and decreased hearing were the most common symptoms in both groups. Specifically, ear discharge was reported in 82.86% of middle-aged patients and 91.43% of younger patients, while decreased hearing was reported in 74.28% of middle-aged patients and 65.71% of younger patients. Other symptoms, such as postauricular swelling, earache, tinnitus, facial weakness, dizziness, and fever, were less frequently observed and showed no statistically significant differences between the groups (Table 2).

**Table 2:** Symptoms seen in middle-aged and younger patients

Symptom	Middle-aged patients	Younger patients	p-value
Ear discharge	29 (82.86%)	32 (91.43%)	1.0000
Decreased hearing	26 (74.28%)	23 (65.71%)	1.0000
Postauricular swelling	1 (2.86%)	1 (2.86%)	1.0000
Earache	2 (5.71%)	1 (2.86%)	1.0000
Tinnitus	2 (5.71%)	0	1.0000
Facial weakness	1 (2.86%)	0	1.0000
Dizziness	2 (5.71%)	0	1.0000
Fever	1 (2.86%)	1 (2.86%)	1.0000

In both middle-aged and younger patients, canal wall up mastoidectomy was more common than canal wall down mastoidectomy. However, compared to younger patients, canal wall down mastoidectomy was more common in middle-aged patients, and canal wall up mastoidectomy was more common in younger patients compared to middle-aged patients. The difference in mastoidectomy procedures was not statistically significant, with a p-value of 0.192 (Table 3).

**Table 3:** Surgical procedures in middle-aged and younger patients CWU, canal wall up; CWD, canal wall down

Surgical procedure	Middle-aged patients (no. of cases)	Younger patients (no. of cases)	p-value
Mastoidectomy by CWU	22 (63%)	27 (77%)	0.0268
Mastoidectomy by CWD	13 (37%)	8 (23%)	1.0000

The postoperative audiological gain in middle-aged patients after three, six, and nine months is detailed in Table 4.

**Table 4:** Postoperative audiological gain in middle-aged patients CWU, canal wall up; CWD, canal wall down; AB, air-bone

Surgical procedure	Time point	Mean preoperative AB gap (dB)	SD (±)	Mean postoperative AB gap (dB)	Mean audiological gain (dB)	SD (±)
CWU	3 months	31.67	3.37	39.97	8.30	3.06
	6 months	31.67	3.37	39.77	8.10	3.19
	9 months	31.67	3.37	39.87	8.20	3.04
CWD	3 months	33.52	3.12	39.92	6.40	3.43
	6 months	33.52	3.12	39.64	6.12	3.55
	9 months	33.52	3.12	39.62	6.10	3.76
Total (CWU + CWD)	3 months	32.36	3.36	39.95	7.59	3.28
	6 months	32.36	3.36	39.68	7.32	3.42
	9 months	32.36	3.36	39.74	7.38	3.43

The postoperative audiological gain in younger patients after three, six, and nine months is presented in Table 5. The middle-aged patients showed a larger preoperative AB gap (32.36 ± 3.36) than the younger patients (27.90 ± 2.43). This difference was statistically significant with a p-value of <0.00001.

**Table 5:** Postoperative audiological gain in younger patients CWU, canal wall up; CWD, canal wall down; AB, air-bone

Surgical procedure	Time point	Mean preoperative AB Gap (dB)	SD (±)	Mean postoperative AB gap (dB)	Mean audiological gain (dB)	SD (±)
CWU	3 months	27.76	2.64	35.28	7.52	2.41
	6 months	27.76	2.64	35.06	7.30	2.54
	9 months	27.76	2.64	34.96	7.20	2.65
CWD	3 months	28.36	1.51	34.62	6.26	2.85
	6 months	28.36	1.51	34.16	5.80	3.41
	9 months	28.36	1.51	34.26	5.90	3.54
Total (CWU + CWD)	3 months	27.90	2.43	35.13	7.23	2.53
	6 months	27.90	2.43	34.87	6.97	2.77
	9 months	27.90	2.43	34.80	6.90	2.86

After three, six, and nine months post-surgery, the overall improvement in the AB gap (mean audiological gain) was higher in middle-aged patients compared to younger patients. However, the difference in the mean audiological gain was not statistically significant, with p-values at 0.3034, 0.3271, and 0.2923, respectively. The audiological gain demonstrated a slight decrease over time in both groups.

## Discussion

In this study, we aimed to compare the outcomes of tympanoplasty with mastoidectomy in middle-aged and younger patients diagnosed with chronic otitis media, with or without cholesteatoma. The primary focus was on evaluating differences in audiological improvement between the two age groups.

In our study, the majority of cases were from rural areas and belonged to low socio-economic strata. This is in concurrence with the study results of Islam et al. who also found a majority of this disease prevalence in rural areas [9]. Rao et al. found that the peak incidence of the disease was between 21 and 30 years of age (45.2%) [2].

The clinical symptoms, particularly, ear discharge and decreased hearing were the most common presentations in both groups. The high prevalence of ear discharge in both groups (82.86% in middle-aged patients and 91.43% in younger patients) highlights the chronic nature of the disease and its impact on quality of life. These findings are consistent with previous studies that identified ear discharge as a primary symptom of COM. Despite the slight difference in percentages, the statistical analysis showed no significant difference between the groups (p = 1.0000).

The overall postoperative mean audiological gain in the middle-aged patients was 7.59 ± 3.28 dB at three months, 7.32 ± 3.42 dB at six months, and 7.38 ± 3.43 dB at nine months; in younger patients, it was 7.23 ± 2.53 dB at three months, 6.97 ± 2.77 dB at six months, and 6.90 ± 2.86 dB at nine months. Although the postoperative mean audiological gain was slight more in the middle-aged patients, the difference was not statistically significant with p-values of 0.30, 0.33, and 0.29 at three, six, and nine months, respectively. This is in concurrence with the study of Ahn et al. who also found that there was no significant difference in the improvement of the AB gap between the two groups, although both preoperative and postoperative AB gaps in the elderly patients were significantly larger than those in the younger patients [5].

In both groups, the postoperative mean audiological gain was more in those patients who underwent tympanoplasty with canal wall up mastoidectomy. The results were consistent with those of Hirsch et al., who reported better hearing outcomes with canal wall up mastoidectomy [10]. Albu et al. also demonstrated that postoperative auditory results significantly improved in canal wall up mastoidectomy cases when compared to open technique cases [11]. Wetmore et al. found in a series of 161 patients with cholesteatoma that the mean pure tone average remained unchanged after surgery. They concluded that canal wall up and canal wall down mastoidectomy procedures had no influence on the hearing outcome [12].

The results of hearing reported in the literature are contrasting. In fact, Paparella et al. reported better results with canal wall up mastoidectomy [13,14]. Karmarkar et al. did not find significant differences between canal wall up and canal wall down mastoidectomy procedures [15]. Tos and Lau were unable to demonstrate a significant difference in the hearing outcomes of canal wall up and canal wall down mastoidectomies [16].

Audiological improvement, measured as the mean audiological gain, demonstrated a slight decrease over time in both groups. In younger patients, the mean audiological gain reduced from 7.23 dB at three months to 6.90 dB at nine months. In middle-aged patients, the mean audiological gain decreased from 7.59 dB at three months to 7.38 dB at nine months. These findings suggest that while initial improvements in hearing are achieved, maintaining these gains over time can be challenging.

The research highlighted that patients aged 21-40 years exhibited the best outcomes in terms of graft uptake and hearing improvement. In contrast, patients younger than 20 years and older than 40 years experienced poorer outcomes. This trend aligns with previous studies that also noted age as a pivotal factor influencing tympanoplasty results. For instance, Tos and Lau reported that the youngest patients, particularly those under 10 years, demonstrated better results in dry ear conditions compared to older patients with discharging ears. Similarly, Emmett found that age significantly affected the success rate of tympanoplasty, with older patients generally having poorer outcomes. However, in our study, we did not find any significant difference in audiological outcomes [17].

This study has certain limitations that should be considered. Firstly, the sample size was relatively small, which may limit the generalizability of the findings to a broader population. Additionally, the postoperative follow-up period was short, restricting the ability to assess long-term audiological outcomes and potential complications. Future studies with larger sample sizes and extended follow-up durations are recommended to provide more robust and comprehensive insights into the audiological outcomes of tympanoplasty with mastoidectomy in middle-aged and younger patients with chronic otitis media.

## Conclusions

Middle-aged patients with COM showed a significantly larger preoperative AB gap than younger patients. Audiological improvement, measured as the mean audiological gain, demonstrated a slight decrease over time in both groups. However, there was no significant difference in the improvement of the AB gap between middle-aged and younger patients. These findings suggest that tympanoplasty with mastoidectomy is an equally effective surgical intervention for improving hearing outcomes in both age groups, irrespective of the initial AB gap. Further studies with larger sample sizes and longer follow-up periods are needed to validate these results and explore additional factors that may influence long-term audiological outcomes.
